# Governing synthetic biology and artificial intelligence (AI) convergence: emerging biosecurity priorities for Africa

**DOI:** 10.3389/fbioe.2026.1834976

**Published:** 2026-08-28

**Authors:** Keletso Masisi, Marie Atsama Amougou, Ilodia Amilcar Zacarias, Ketan Thorat, Robert Banai, Janes Mokgadi, Brett Edwards

**Affiliations:** 1 Department of Biological Sciences and Biotechnology, Botswana International University of Science and Technology, Palapye, Botswana; 2 Research Center on Emerging and Re-emerging Diseases, Institute of Medical Research and Medicinal Plants Studies, Yaounde, Cameroon; 3 360 Platform for Africa, Maputo, Mozambique; 4 Institute for Stem Cell Science and Regenerative Medicine, Bengaluru, India; 5 World Health Organization, Kinshasa, Democratic Republic of the Congo, Yaounde, Cameroon; 6 Chemical, Biological, Radiological and Nuclear Weapons Management Authority, Gaborone, Botswana; 7 Politics, Languages & International Studies, University of Bath, Bath, United Kingdom

**Keywords:** Africa, artificial intelligence, biosecurity, governance, synthetic biology

## Abstract

The rapid convergence of synthetic biology and artificial intelligence is reshaping the biosecurity landscape, yet governance approaches have not kept pace with the realities of digitally mediated biological research, particularly in African contexts. Existing global discourse has largely centred on high-level frameworks and treaty-based mechanisms, with comparatively less attention to how governance is operationalised within institutional systems. This paper addresses this gap by introducing and discussing structural exposure points defined here as systemic governance conditions that increase the likelihood that emerging technological capabilities will outpace oversight. Through an analysis of African biosafety regulatory bodies and national frameworks, the study identifies key exposure points, including fragmented institutional mandates, uneven regulatory and technical capacity, dependence on external technological infrastructure and limited integration of AI literacy within biosafety and biosecurity systems. Building on this analysis, the paper proposes a set of pragmatic and context-sensitive governance priorities, including embedding AI considerations within existing biosafety and research review processes, adopting proportionate risk-based oversight approaches, strengthening cross-disciplinary capacity and establishing baseline expectations for DNA synthesis screening. It further advances an adaptive governance approach centred on training key personnel, integrating knowledge across institutional systems, strengthening coordination functions and enabling continuous policy learning through feedback. By shifting the focus from regulatory expansion to institutional integration, this paper intends to offer a feasible pathway for strengthening biosecurity governance in Africa hence helping support responsible scientific innovation while addressing the evolving risks of bio-digital convergence.

## 1 Introduction

The fields of synthetic biology (SynBio) and artificial intelligence (AI) represent two emergent techno-scientific domains. Synthetic biology has, since at least 2006, been understood as an area of innovation based on advances in genetic engineering and computing, which has focused on developing new technology platforms and applications (Carter and Warner, 2018; De Haro, 2024; Mohseni and Ghorbani, 2024; Zhang et al., 2024). In recent years, the field of AI has matured and has produced a suite of capabilities which are being rapidly integrated into research and development, not least in fields such as synthetic biology (Carter and Warner, 2018; Zhang et al., 2024). Advances in genome editing such as the use of CRISPR(Clustered Regularly Interspaced Short Palindromic Repeats), AI-driven protein modelling, *de novo* organism design and automated laboratory systems over the past decade have profoundly changed the way biological systems are engineered, monitored and controlled (Carter and Warner, 2018; Mohseni and Ghorbani, 2024; Zhang et al., 2024; Dong et al., 2025). The ease of access to AI platforms has made it easy to analyse biological datasets such as genome sequences, protein folding mechanisms and vaccine development. Globally, the use of SynBio and AI has had direct positive implications for public health, agriculture and industrial biotechnology. As previously highlighted in discussions surrounding the Biological Weapons Convention (BWC), these developments expand both beneficial applications and dual-use risks. For example, the AI driven SynBio developments can increase the ease of cyber-biothreats that may have not been previously possible. These biothreats can lead to weaponization by corruption and weaponization by denial, defined respectively as the creation of a bioweapon by a cyber tool and the removal of a beneficial biological entity such as a vaccine from the population (Elgabry and Johnson, 2024). However, as demonstrated during recent public health crises, technological innovation often evolves faster than the frameworks intended to govern it (Mwapagha, 2023; Agboli et al., 2025; Muoneke et al., 2025). This similar pattern is noticeable with the development of SynBio and AI which have raised dual use concerns. For example, AI systems have made it possible to automate design-build-test cycles, while synthetic biology platforms and cloud-based services enable biomanufacturing and distribution of biomaterials such as DNA (Agboli et al., 2025; Ochola, 2025). For example a planning agent such as CRISPR-GPT which allows easier and automated design around genome editing can be easily accessible and potentially mishandled if not regulated. Although the combination of SynBio and AI holds opportunities it also poses risks which can cause barriers to beneficial innovation while blurring the boundaries among laboratory safety, biosecurity, and digital governance (Timcke, 2025). One of the main concerns with the use of AI is its availability and ease of access that can be unregulated hence posing a risk of being used by ungoverned non-state actors, small laboratory groups and individuals. In addition more specialised AI tools using large language models (LMs) are used for designing proteins and genomes. This can speed up the design-build-test-learn cycle that is used in SynBio hence raising concerns of the tools being adopted for pathogen related research (Hynek, 2026). Some analyses have also indicated that present models can offer mechanisms for pathogen design (Sabra et al., 2026). These advancements also raise concerns over regulation with international and regional frameworks.

International biosafety and biosecurity frameworks and treaties such as the BWC, WHO Global Guidance Framework for the Responsible Use of Life Sciences, Global Health Security Agenda (GHSA), mostly focus on stopping the production and stocking of biological weapons, ethics control and laboratory safety practices (Sabra et al., 2026). However, there is less on the emphasis and governance of intangible tools such as SynBio and AI hence leaving these topics mostly under governed. Other existing regulations such as the UNESCO’s AI ethics guidance, the EU Act and the International Health Regulations (IHR) still lack in the detailed strategies on the governance of AI and SynBio (EvalCommunity Academy, 2025). Hence, a more encompassing governance framework that is dedicated to more complex AI-SynBio technologies is still unavailable (Groff-Vindman et al., 2025). This then raises the problem that may affect governance if there are no proper regulations in place to provide oversight of these technologies. This challenge is further increased by the fact that both the technologies and their synergy are rapidly evolving, therefore making it hard to keep track of their developments (Ibeneme et al., 2021).

On the other hand treaties like the BWC possess a comprehensive scope however, its effective implementation relies on a regime capable of adapting to rapid advancements in science and technology (Townsend et al., 2023). In the absence of a verification regime comparable to the Chemical Weapons Convention, addressing the governance of AI driven mechanisms in synthetic biology requires continuous and multilevel approaches. This necessitates identifying trends of concern, forming political consensus on areas for action, and implementing these through multilevel governance frameworks. Currently, there is a disconnect between global policy and practical implementation, as comparatively less attention has been given to how governance principles are operationalized within institutional systems, particularly concerning routine risk assessment, regulatory coordination, and daily oversight mechanisms (Hynek, 2026). These challenges have been more prevalent across Africa, where the convergence poses a unique governance challenge. While biotechnology ecosystems are growing through academic research, One Health platforms, genomic surveillance networks, and innovation startups, regulatory authorities often operate separately. A limited number of trained inspectors, uneven laboratory accreditation, constrained cyberbiosecurity infrastructure, and reliance on external digital platforms add layers of complexity to national and regional governance (Ibeneme et al., 2021; Frimpong, 2025; Owor et al., 2025). These concerns are particularly relevant in Africa, where investments in molecular diagnostics, genomic sequencing and digital health have increased following major epidemics such as Ebola and COVID-19 (Asin-Garcia et al., 2025; Dong et al., 2025; Facini, 2025).

This gap is further compounded by existing challenges, including fragmented regulatory mandates and limited integration between biosafety, biosecurity and emerging digital governance frameworks (Groff-Vindman et al., 2025; Osman and Abdullah, 2026). In Africa, different continental biosafety and biosecurity bodies have made progress in being able to identify the threats, however, many frameworks still recognize threats as material-based (ASLM, 2025; Dunne et al., 2026). As an example, the emphasis on auditing physical infrastructure instead of a combined digital infrastructure where dual use can occur using genetic sequences. In addition, most bilateral biosafety networks have foundational alignment with frameworks such as the Cartagena Protocol which has a legally binding capacity to “Living Modified Organisms” (LMO’s) (Bond and Scott, 2020; Akpoviri et al., 2023); therefore, leaving out any intangible threats such as generative AI algorithms that may be used maliciously. While discussions around treaty interpretations and verification are essential for strengthening international norms (Qin et al., 2026), comparatively less attention has been given to how governance principles relating to AI and SynBio are operationalized within institutional systems in the region, particularly in relation to routine risk assessment, regulatory coordination and day-to-day oversight mechanisms. This, in turn, creates a disconnect between global policy and practical implementation.

### 1.1 Continental biosafety bodies

Previously, African governance structures have governed advances in technology such as AI and advances in biotechnology such as Synbio separately (Sabra et al., 2026). Another common limitation with the governance of the technologies has been the reliance on generalised frameworks that view digital data and “live” biological risks as separate entities. However, the combined risk of AI and Synbio completely nullifies this view, indicating that the use of both technologies in combination can be a risk on its own (Kilungu and Tombe, 2026; Sabra et al., 2026). To show this governance gap, Table 1 shows some of the existing biotechnology, biosafety and biosecurity framework bodies in the continent and highlights some of the gaps regarding AI-Synbio governance.

**TABLE 1:** Regional technical and accredited biosafety bodies’ frameworks and their governance gaps on AI-SynBio convergance.

Continental body	Framework/standard/initiative	Governance gaps regarding AI-SynBio	References
Africa CDC	Biosafety and Biosecurity Initiative (BBI) strategic roadmap 2026–2030 Africa CDC High-Containment Laboratory (HCL) specialized mandate (2026)	Certification tracks are entirely tailored to physical containment engineering and laboratory safety No certified training tier or operational framework for auditing generative algorithmic bio-design or dual-use sequence screening Lack of specialized computational biology frameworks or localized server-auditing tools needed to monitor digital sequence transfers or automated DNA synthesis	Frimpong (2025)
ASLM (African Society for Laboratory Medicine)	ASLM strategy 2021–2025 laboratory compliance monitoring frameworks	Audit diagnostic and physical laboratories Does not encompass auditing cloud-based synthetic biology operations where automated design software can bypass traditional diagnostic physical pipelines entirely	Huigang et al. (2022), Owor et al. (2025)
AfBNet (African Biosafety Network)	AfBNet constitution and professional code of practice	Bound to traditional biosafety levels (BSL-1 to BSL-4) Limited mitigation protocols for the digital dissemination of synthetic biology dual-use	Trump et al. (2026)
AUDA - NEPAD (African Union Development Agency)	AI continental roadmap -2025–2030	Provides a roadmap for digital industrilization Establishment of continental AI certification standard Lacks biosecurity guidelines for sequence-screening Failure to account for cyber-biosecurity or cross-border digital sequence information oversight	de Lima et al. (2024)

### 1.2 Current suggestions for AI-SynBio governance

A number of governance approaches have been proposed to govern the developments associated with SynBio and the related AI technologies (Table 2). Although no dedicated international framework currently exists to solely govern the aforementioned technologies, several recurring themes have emerged within the literature. One prominent proposal involves strengthening science and technology review mechanisms to enable continuous monitoring of rapidly evolving capabilities in AI-assisted biological design. Related recommendations emphasize anticipatory governance approaches that can identify emerging risks before they become embedded within research and innovation systems (Hynek, 2026; Sabra et al., 2026). Other proposals have focused on strengthening oversight of the design phase of biological research through enhanced DNA synthesis screening, improved verification procedures and greater transparency regarding the use of computational tools in biological design workflows (Agboli et al., 2025; Ochola, 2025). Increasing attention has also been given to the governance of cloud laboratories, automated experimentation platforms, and AI-enabled biological design systems, reflecting concerns that biosecurity risks may increasingly originate from digitally mediated stages of research rather than exclusively from laboratory-based activities (Frimpong, 2025).

**TABLE 2:** Suggested AI-SynBio governance strategies.

Framework/treaty/initiative	Primary scope	Suggested adaptation for AI-Synbio governance	References
Biological Weapons Convention (BWC)	Disarmament and prohibition of biological weapons	Clarify that AI-enabled biological design and automated experimentation fall within BWC obligations; develop living science-and-technology review processes that explicitly consider AI design tools and DNA synthesis workflows	Huigang et al. (2022), Trump et al. (2026)
International Health Regulations (2005) and WHO laboratory biosafety/biosecurity guidance	Global health security, surveillance, and laboratory systems	Integrate AI-enabled synthetic biology scenarios into national preparedness plans and core capacity assessments; include AI-assisted design, cloud services, and DNA synthesis supply chains in national biorisk assessments, exercises, and laboratory guidance	International Health Regulations (2005)
African Union (AU) and Africa CDC health-security and laboratory frameworks	Regional health security and biosafety/biosecurity systems	Use AU/Africa CDC processes to develop regional guidance on AI-SynBio governance, including model policies, peer learning, shared incident-reporting templates, and harmonized expectations for DNA synthesis screening and cyber-biosecurity across member states	Standley (2025), Mutesi et al. (2025)
EU artificial intelligence Act and related AI governance frameworks	Horizontal regulation of AI using a risk-based approach	Treat AI systems used for biological design, optimization, or synthesis as high-risk; require pre-deployment risk assessment, documentation of training data and model capabilities, logging of bio-relevant outputs, and independent oversight for sensitive AI-SynBio applications	Ayana et al. (2024), Victor (2025)
UNESCO recommendation on the ethics of AI and OECD principles on AI and synthetic biology	Normative guidance for trustworthy and responsible AI and SynBio	Use these as normative anchors for AI-SynBio by requiring context-sensitive benefit-sharing, inclusion of African stakeholders in standard-setting, attention to digital colonialism and technological sovereignty, and explicit consideration of equity in access to AI-SynBio capabilities	EvalCommunity Academy (2025)

Several studies advocate for adaptive and risk-proportionate governance models that integrate biosafety, biosecurity, cybersecurity, and AI governance considerations within a common oversight framework. Capacity building, interdisciplinary training, responsible innovation practices and stronger collaboration between biological scientists, AI developers, regulators, and policy communities have similarly been identified as important components of future governance systems (Owor et al., 2025; Sabra et al., 2026), However, much of this discussion remains conceptual and is primarily focused on identifying potential governance pathways rather than establishing internationally agreed standards.

While the governance strategies are forward looking, it may be difficult to implement them in some states that are currently experiencing institutional biosafety and biosecurity challenges. Additionally regional biosafety and biosecurity bodies are still lagging behind on how to implement the governance of AI assisted Synbio developments. Therefore to make the adoption easier it may be essential to highlight and address the underlying governance issues first. Therefore, this paper explores how emerging and suggested governance approaches for SynBio-AI convergance may be influenced by the institutional realities in African states in which they may be implemented. It argues that the effectiveness of proposed governance mechanisms will largely depend on underlying current governance conditions that may hinder implementation.

### 1.3 Methodological approach

To examine these challenges, the paper discusses the concept of structural exposure points, defined here as systemic governance conditions that increase the likelihood that rapidly evolving technological capabilities outpace existing oversight systems. We use African regulatory contexts as a case study and conduct analyses on how factors such as fragmented oversight mandates, uneven institutional capacity, dependence on external technological infrastructure and limited integration of AI expertise within biosafety systems may create governance challenges for AI and Synbio technologies. The paper further examines how these structural exposure points may affect the implementation of governance approaches currently proposed in the literature. Building on this analysis, it proposes pragmatic and context-sensitive governance priorities aimed at strengthening institutional preparedness, regulatory coordination and workforce development while supporting the safe and beneficial use of emerging bio-digital technologies within the region.

This paper further adopts a qualitative policy and governance review of the continent’s most prominent biosafety governing bodies in order to identify governance gaps. African countries that have developed AI, synbio and biosafety strategies and frameworks are also evaluated. To do this, peer-reviewed literature was combined with analysis of publicly available legislation, national policies and regional governance frameworks relevant to biosafety, biosecurity, artificial intelligence and synthetic biology. Documents were purposively selected based on their relevance to AI governance, biosafety and biosecurity regulation, health security, laboratory governance and emerging biotechnology within African contexts. The review considered continental frameworks together with selected national policies from countries that have developed AI strategies, biosafety legislation or related governance instruments. These documents were analysed to identify recurring governance characteristics and implementation challenges that may influence future governance of AI and Synbio in the region.

## 2 Existing national governance instruments relevant to AI and Synbio

### 2.1 Artificial intelligence governance

Most African countries are increasingly recognising artificial intelligence as a strategic technology requiring dedicated policy attention. As an example the South Africa’s Draft National Artificial Intelligence Policy Framework places considerable emphasis on ethical AI deployment, public-sector accountability, algorithmic transparency and inclusive socio-economic development. Nigeria’s National Artificial Intelligence Strategy (2024) identifies AI as a driver of national competitiveness through investment in local computing infrastructure, foundational model development and sectoral innovation (Mienye et al., 2024; Kwarkye, 2025; Chege, 2026). Kenya’s National AI Strategy (2025–2030) also adopts a flexible governance approach centred around innovation-friendly oversight and private-sector participation (Muchiri, 2025). Comparable priorities are also noticed within Botswana’s SmartBots National Strategy which is supported by the United Nations Economic Commission for Africa and accompanying Digital Services and Cybersecurity Acts, Ethiopia’s National Artificial Intelligence Policy, and Egypt’s National Artificial Intelligence Strategy, also position AI as an enabling technology for digital transformation and economic diversification (Mienye et al., 2024; Kwarkye, 2025; Chege, 2026).

Across the strategies and frameworks examined, AI is predominantly framed as a digital technology requiring governance to promote innovation, economic growth, ethical deployment, algorithmic fairness and data protection. However, there is a continued lack of noticing the implications of AI assisted biological research and its associated risks. An example is the South Africa’s Draft AI Framework, that establishes principles for responsible AI deployment and algorithmic accountability but does not explicitly consider AI systems capable of generating biological designs or supporting synthetic biology. Nigeria’s National Artificial Intelligence Strategy similarly prioritises sectoral deployment across agriculture, healthcare, education and public administration but contains no dedicated provisions addressing the governance of dual-use computational biology or AI-assisted pathogen design. Again, Kenya’s AI Strategy promotes adaptive regulation through regulations intended to help technological innovation, yet these mechanisms remain largely focused on software innovation and digital entrepreneurship excluding computational biological engineering. Likewise, Ethiopia’s National Artificial Intelligence Policy introduces principles for trustworthy AI while emphasising applications in health, national defence and public administration. However, oversight mechanisms remain centred on software governance and algorithmic deployment rather than AI-enabled biological research (Hanna et al., 2025; Wheeler, 2025; Sule, 2026).

This pattern becomes more apparent when considering the treatment of data governance within other reviewed national strategies. South Africa’s Protection of Personal Information Act (POPIA), Nigeria’s Data Protection Act, Kenya’s Data Protection Act, Egypt’s Personal Data Protection Law, Uganda’s Data Protection and Privacy Act, and Ghana’s Data Protection Act all establish comprehensive legal safeguards governing the collection, processing, transfer and storage of personal information using digital technologies like AI (Munung et al., 2024; Ogbodo et al., 2025). These indicate important advances in protecting digital rights and public trust in emerging technologies. However, they consistently conceptualize data as information requiring protection primarily for reasons of privacy, ownership, security or commercial value (Romansky and Noninska, 2020; Misra et al., 2025). None explicitly differentiates conventional digital information from biological sequence data, genomic datasets, protein structures or other forms of digital biological information that may simultaneously constitute scientific data and potential sources of biological capability. As synthetic biology increasingly relies on digital sequence information and AI foundation models trained on large biological datasets, the absence of governance mechanisms recognising the dual-use nature of biological information represents an important regulatory limitation (Hanna et al., 2025; Wheeler, 2025; Sule, 2026). While these data governance frameworks remain appropriate for protecting individual privacy and digital rights, they provide limited guidance regarding the governance of computational tools capable of generating or optimising biologically functional sequences before any physical biological material exists (Trump et al., 2026).

Although this review shows that several African countries have initiated the development of national AI strategies and policy frameworks, AI governance across the continent remains an evolving policy domain. The withdrawal of South Africa’s Draft National Artificial Intelligence Policy prior to its adoption, following concerns over the integrity of its supporting evidence, highlights the complexities associated with developing governance for emerging technologies. This serves as a reminder that effective AI governance depends not only on the existence of policy initiatives but also on the institutional expertise, technical credibility, and evidence-based processes required to support their development and implementation (Thaldar et al., 2025; Chege, 2026; Nyagoga et al., 2026).

### 2.2 Existing biosafety strategies on emerging technologies

In addition to AI governance, biotechnology and biosafety startegies have expanded over the past decade. For example numerous countries have established comprehensive legal frameworks governing genetically modified organisms, laboratory biosafety, contained use, environmental release and public health protection (Njagi et al., 2026). These were largely driven by obligations under international treaties such as the Cartagena Protocol on Biosafety and implementation of the BWC. Some countries now have governing aspects of modern biotechnology, supported by their national authorities, institutional biosafety committees and national advisory boards. For example, Nigeria has adopted one of the most progressive legislative approaches by explicitly recognising gene editing and synthetic biology within the National Biosafety Management Agency (Amendment) Act of 2019. The legislation expands the authority of the National Biosafety Management Agency (NBMA) to regulate emerging biotechnology applications beyond conventional genetically modified organisms, demonstrating a forward-looking appreciation of evolving biological technologies (Ongu et al., 2023; Zawedde et al., 2025; Osman and Abdullah, 2026). Similarly, South Africa’s Genetically Modified Organisms Act, together with the National Environmental Management: Biodiversity Act (NEMBA), establishes robust regulatory oversight for the development, production, importation, environmental release and commercialisation of genetically modified organisms. Kenya’s Biosafety Act (2009) provides comprehensive statutory authority to the National Biosafety Authority for regulating contained use, importation, transit and environmental release of GMOs, while Ghana’s Biosafety Act (Act 831) establishes comparable oversight through the National Biosafety Authority and Technical Advisory Committee. Uganda’s National Biosafety Act (2017), Ethiopia’s Biosafety (Amendment) Proclamation, Zambia’s Biosafety Act and Egypt’s ministerial biosafety directives similarly provide structured regulatory systems governing biotechnology research, confined field trials, environmental release and transboundary movement of modified organisms. Botswana’s Biological and Toxin Weapons (Prohibition) Act complements these biosafety frameworks aswell by strengthening national implementation of BWC obligations through the regulation of controlled biological agents and toxins (Muzhinji and Ntuli, 2020; Ongu et al., 2023; Njagi et al., 2026).

Nevertheless, these aforementioned strategies are still tied to address predominantly physical risks without any regulation on the intangible risks. South Africa’s GMO Act, for example, regulates the production, use and release of genetically modified organisms but provides no equivalent mechanism for evaluating computational sequence design prior to physical synthesis (Muzhinji and Ntuli, 2020). Similarly, Ethiopia’s Biosafety Proclamation defines regulated “transactions” primarily in terms of importation, exportation, transit, contained use and environmental release of modified organisms, thereby assuming that governance begins once biological material enters laboratory or trade environments (Abraham, 2013). Uganda’s National Biosafety Act follows a comparable model by concentrating regulatory oversight on confined field trials and physical biotechnology activities (Njagi et al., 2026)while Zambia’s Biosafety Act similarly focuses on licensing the research, movement, handling and commercial application of genetically modified materials. Egypt’s implementation of Cartagena Protocol obligations continues to emphasise border inspection, quarantine procedures and physical movement of biological materials across national boundaries (Ongu et al., 2023; Njagi et al., 2026). In Cameroon, the intersection of AI and biosecurity remains shaped by a governance landscape still calibrated to physical pathogens and laboratory risks, so that digital biology and AI-based sequence design fall largely outside explicit regulatory oversight, and the dual-use risks of AI in biotechnology are only indirectly managed through traditional biosecurity frameworks and nascent AI strategies focused on digital sovereignty rather than on scrutinising genomic data flows or AI-enabled sequence design prior to physical synthesis (Atem, 2026a; 2026b). These legislative approaches remain entirely appropriate for the governance challenges they were originally designed to address, however, they provide comparatively limited oversight of upstream computational activities such as AI-enables synthetic biology that influence the production of these organisms.

A second recurring observation is the operational focus of existing biosafety institutions. Institutional biosafety committees, national biosafety authorities, and scientific advisory committees remain central to biotechnology oversight across the reviewed countries showing internationally recognised best practice for laboratory governance. However, their mandates and technical expertise continue to be oriented toward conventional biosafety concerns, including laboratory containment, environmental risk assessment, food safety, occupational safety and compliance with biotechnology legislation. This is evident within South Africa’s Advisory Committee established under the GMO Act, Kenya’s National Biosafety Authority, Ghana’s Technical Advisory Committee, Uganda’s National Biosafety Committee and Ethiopia’s National Biosafety Advisory structures where they all provide important scientific oversight for biotechnology activities (Ongu et al., 2023; Zawedde et al., 2025; Njagi et al., 2026). Yet none of them explicitly requires assessment of AI models assisting in biotechnological research, algorithmic sequence optimisation, digital biological datasets or biological foundation models during biosafety review processes. However, this does not imply deficiencies within existing institutions but it reflects the historical reality that most of these oversight systems were established before AI became an integral component of biological research. Additionally, this observation suggests that implementation of emerging governance proposals for AI and Synbio within the continent will benefit from strengthening coordination across existing legislative mandates, regulatory institutions and sector-specific governance systems. This entails extending beyond molecular biology and biosafety to include computational biology, machine learning, cyberbiosecurity and digital systems governance. We therefore conceptualise these governance challenges as structural exposure points. The following section examines each of these structural exposure points in detail and considers their implications for the proposed biosafety review prosses for AI and Synbio in the region.

## 3 Structural exposure points in the governance of SynBio-AI convergence

The structural exposure points detailed hereafter create the systemic conditions under which governance in the bio-digital interface must operate-. While these conditions set the broader context, they inevitably manifest as operational limitations in the mechanisms through which oversight is implemented. The following section examines these specific governance challenges, demonstrating how existing regulatory architectures struggle to adapt to the complexities introduced by AI-enabled biological design in the region (Choge et al., 2026).

There has been limited attention to how the structural characteristics of regulatory systems in low and middle income settings shape bio digital risk. This oversight reflects a broader tendency in biosecurity governance to ignore how policy is shaped by specific political and institutional contexts rather than purely technical requirements, a theme central to understanding how security and emerging biotechnology are socially constructed governance systems at the national, regional and global level (Edwards, 2019). In Africa, the risk associated with SynBio and the related AI technologies unfolds within institutional, infrastructural and capacity conditions that differ from those assumed in many global debates (Victor, 2025). Understanding these structural exposure points is essential for preparing and designing governance approaches that are both proportionate and operationally feasible. The current biosafety and biosecurity landscape has expanded based on the growing wet lab biotechnology investment and less on genomic surveillance initiatives and *in silico* biotechnology advancements. Over the past two decades, many sub-Saharan African countries have adopted or updated biosafety and biosecurity policies and legal frameworks, often in response to international obligations, donor-supported health security programs and high-consequence outbreaks such as Ebola virus disease and COVID-19. These instruments typically cover laboratory biosafety, occupational health and safety, public health emergency preparedness and, in some cases, agricultural or environmental biosafety. The COVID-19 pandemic further catalyzed investment in molecular diagnostics, genomic sequencing and laboratory capacity, including expanded diagnostic platforms, point-of-care tools (Ibeneme et al., 2021) and next-generation sequencing, supported by initiatives such as the Africa CDC Laboratory Network and the African Union health security agenda. Implementation, however, remains uneven due to institutional arrangements, financing and, program resourcing varying widely, with some countries operating standards-aligned systems while others are at early stages of policy development (Frimpong, 2025). This then indicates the need for flexible, status-appropriate approaches to governance strengthening to level the playing field and promote a more standardized biosafety and biosecurity governance improvements especially for AI and Synbio. This approach may be helpful in positioning many African nations to be better adopted and ready for future AI-Synbio governance frameworks.

### 3.1 Regulatory fragmentation across bio-digital domains

Regulatory oversight relevant to SynBio and AI is typically distributed across multiple sectoral authorities. Biosafety frameworks may be administered under environmental ministries while biosecurity responsibilities often sit within defence or national security structures. Cybersecurity and digital governance fall under communications or ICT authorities; and emerging AI policy initiatives may be housed within innovation or technology portfolios (Samori et al., 2023). The comparative national reviews (Section 2.1) indicated that responsibility for governance relevant to AI and synthetic biology is distributed across multiple institutions. Biosafety legislation is typically administered through national biosafety authorities or environmental ministries, while AI governance, where present, falls under digital transformation or ICT ministries. These mandates have evolved independently, reflecting the need to combine any developments with emerging technologies in synthetic biology across the region. The need for an integrated risk assessment workflows involving synthetic biology and AI to allow safe use without compromising biosecurity is crucial (De Haro, 2024). In addition, institutional review mechanisms rarely integrate these dimensions within a single evaluative framework. For example, biosafety committees may assess containment levels and pathogen classifications without systematically considering the role of algorithmic design tools or the provenance of digital models. Conversely, AI governance initiatives often prioritize data protection, fairness, and transparency, with comparatively less emphasis on systematically incorporating biological misuse considerations. This misalignment can indicate a coordination deficit within the bio–digital interface, where overlapping technological domains are governed through separate and insufficiently connected oversight systems.

The fragmentation can also be noticed in the reporting of cyber bio-incidents. Events involving unauthorized access to biological design software, manipulation of digital sequence data, or compromise of laboratory automation platforms may overlap the mandates of cybersecurity and biosafety authorities (Samori et al., 2023). In many contexts, reporting pathways for digital breaches are institutionally separate from those for laboratory biosafety incidents. This division can lead to delayed notification, incomplete information exchange, or jurisdictional ambiguity in managing hybrid cyber-bio events. The absence of integrated reporting protocols can limit the ability of authorities to generate comprehensive situational awareness. Developing coordinated reporting frameworks that encompass both biological and digital dimensions would strengthen early detection and response capacity.

While international forums address developments in science and technology, structured regional coordination mechanisms within Africa for monitoring AI-enabled dual-use developments remain limited. National science advisory processes vary in scope and resources, and systematic information-sharing on emerging bio–digital tools is not uniformly institutionalized (Ibeneme et al., 2021). In many African countries, research governance frameworks such as institutional ethics committees, biosafety committees, or national scientific councils are still consolidating and may not yet systematically assess dual-use or misuse risks associated with advanced biological design tools (Alley et al., 2019; Wheeler, 2025; Trump et al., 2026). This comes with limited explicit reference to SynBio, AI-enabled design tools, computational workflows, or the specific risks posed by DNA sequence synthesis. This can result in a significant gap between the actual risk profile of SynBio-AI projects and the formal oversight mechanisms applied, particularly in academic or private-sector settings. Researchers and review committees may also lack clear guidance on how to assess dual-use risks or what additional safeguards are needed (Hoffmann et al., 2023; Xiang et al., 2026).

### 3.2 Cross-border dependence on external technological infrastructure

A further structural feature shaping exposure is reliance on external digital and biological infrastructure. Many research institutions across the continent depend on internationally hosted cloud platforms for computational modelling, machine learning tools and biological data storage (Ayana et al., 2024). DNA synthesis services are frequently outsourced to providers located in other states. Critical reagents, laboratory equipment and proprietary software are often imported. This global integration enables participation in research but complicates governance. For example screening standards for DNA orders may be determined by foreign commercial entities and AI models (Salami, 2024) are defined and trained by technology providers whose compliance regimes operate outside domestic regulatory control (Salami, 2024). Smaller private laboratories or early-stage startups may operate under general public health regulations without specialized guidance tailored to emerging technologies. In addition, cross-border mobility of researchers and collaborative networks may further diversify operational practices. Where there is use of both AI and Syn-Bio, biological design may occur digitally within one country, synthesis in another and experimental validation in a third. Such distributed workflows blur lines of regulatory authority in most African states. For states with high degrees of infrastructural interdependence, effective biosecurity governance must therefore incorporate mechanisms for cross-border coordination, verification of supplier standards and clarity regarding digital data governance.

### 3.3 Limited integration of AI literacy into biosafety and biosecurity practice

A critical yet underexplored dimension of governance is the level of AI literacy within existing biosafety and biosecurity institutions. Traditional biosafety training emphasises pathogen classification, containment levels, waste management and laboratory procedural compliance. AI governance discussions, in contrast, tend to centre on algorithmic transparency, bias mitigation, and data ethics. These knowledge domains have largely evolved in parallel and their combination can help overcome the dual use problem.

There is more need for evaluators who understand how computational models can influence biological design choices, reduce expertise thresholds, or automate iterative experimentation. Without foundational literacy in model capabilities, training data considerations and digital workflow architectures, biosafety reviewers may be unable to fully assess the implications of AI-assisted projects. Oversight may then focus primarily on downstream laboratory risks while underestimating upstream design-phase vulnerabilities. The need to fill this gap has been emphasised for African countries in order to address the AI ecosystem with some research groups even developing language models using local languages (Pantserev, 2025). If biosafety institutions do not internalise this shift, risk assessments may not fully capture the expanded design space enabled by AI. Strengthening AI literacy within biosecurity governance is therefore necessary for meaningful oversight of contemporary life sciences research.

This challenge has also led to asymmetric oversight capacity in personnel competence relating to AI and synthetic biology. The growth of technical capability has not uniformly been matched by parallel investments in oversight infrastructure. Effective governance of AI enabled research requires evaluative personnel competence in computational workflows, digital traceability and model-based experimentation (Wang and Zhang, 2019; Sun et al., 2022). Yet specialised personnel capable of assessing both advanced molecular biology and AI systems functionality remain limited. National authorities often operate with constrained numbers of inspectors, limited access to advanced genomic forensics and insufficient digital audit systems capable of tracing computational risks. This means technological tools capable of accelerating biological design are increasingly accessible, while the evaluative and monitoring capacity required to supervise their responsible use develops more slowly. This imbalance may not produce immediate regulatory failure, but it can weaken the depth and consistency of risk assessment, particularly in complex or high-consequence research establishments in the region.

## 4 Governance challenges within SynBio-digital interface

The convergence of synthetic biology and artificial intelligence exposes limitations in the operational mechanisms through which oversight is implemented. Existing governance systems were largely designed to supervise laboratory-bound biological research, with clear descriptions of responsibility and containment standards. AI-enabled biological design, however, introduces digitally mediated workflows and multi-actor participation that strain these traditional oversight architectures. The following gaps illustrate areas where current mechanisms are misaligned.

### 4.1 Limited visibility of AI use in biosafety review

Review of biosafety legislation, institutional mandates and available biosafety review guidance across the selected countries indicates that oversight strategies primarily focus on genetically modified organisms, laboratory containment, environmental release and research ethics (Table 1). None of the reviewed governance instruments explicitly requires disclosure of AI-assisted biological design, computational sequence optimisation or machine-learning tools used during experimental planning. Institutional biosafety committees (IBCs) and related bodies typically assess research proposals based on organism classification, personnel qualifications and procedural safeguards. Review templates can require less explicit disclosure of AI-assisted design tools, computational model selection, or the degree of automation involved in experimental planning (FAQs on Institutional Biosafety Committee IBC Administration–April 2024, n.d.). Consequently, upstream design decisions such as sequence optimisation or functional prediction generated by machine learning systems may remain outside formal scrutiny (Samori et al., 2023). This absence of structured disclosure mechanisms creates a procedural blind spot. Where AI systems materially influence experimental design, risk assessment that focuses exclusively on downstream laboratory practices may fail to capture the full spectrum of design-phase vulnerabilities. Integrating rigorous AI usage reporting requirements into biosafety review processes represents a necessary adjustment to align oversight with contemporary research review (Tuscano and Disso, 2026). In low and middle income countries the limited AI use within biosafety review processes and research may be compounded by broader governance and capacity constraints affecting the integration of digital technologies into health and research systems such as anti microbial resistance (AMR) surveillance. For example a study by (Lyimo, 2026) focusing on combating AMR in Africa indicated that in most African countries bioinformatics analysis and trainings remains fragmented and challenging to access with approximately 10% of researchers only having access to high performance computing facilities (HPC). It is further suggested that introducing AI such as deep learning can assist in automating and analysing large scale genomic datasets using models that were previously difficult to acquire. As AI-enabled tools become increasingly incorporated into genomics, diagnostics, and biomedical research across the region, oversight mechanisms have not consistently evolved to account for the use of computational models within existing biosafety review procedures (Edwards, 2019). Recent discussions on AI governance in Africa have similarly highlighted challenges related to institutional readiness, regulatory coordination, and the adaptation of oversight systems to rapidly evolving technologies.

### 4.2 Inconsistent DNA synthesis screening practices

DNA synthesis screening constitutes a critical preventive control within the design–build pipeline (Wang and Zhang, 2019). However, screening standards vary across commercial providers, and transparency regarding methodologies and databases can be uneven (Gillum and Moritz, 2025). In many African regions, there is no harmonized regulatory requirement mandating verification of screening compliance for imported synthetic sequences. Given that a significant proportion of synthesis services are procured from international suppliers, oversight frequently depends on external corporate policies rather than regionally defined standards. This variability weakens the predictability of preventive safeguards and may create uneven risk thresholds across institutions. Establishing baseline expectations for sequence screening regardless of supplier location would reduce dependence on external partners (Simon, 2025). These governance challenges are further compounded by limited local DNA synthesis capacity and a continued reliance on imported synthesis services and external providers. Emerging analyses indicate that DNA synthesis activity across the continent remains uneven, with much of the market dependent on suppliers outside the region and with limited visibility into the screening standards applied to submitted sequences (Wang et al., 2026). At the same time, studies examining biosecurity practices among DNA providers globally have demonstrated variability in screening approaches, customer verification procedures, and sequence oversight mechanisms (Gillum and Moritz, 2025; Simon, 2025). This creates important governance challenges for African research systems, where local oversight of externally conducted synthesis may be constrained by fragmented regulatory frameworks, limited technical capacity, and the absence of harmonized regional screening expectations. As synthetic biology and AI-enabled biological design become more integrated into research and innovation systems, strengthening regionally aligned approaches to DNA synthesis governance may therefore become increasingly important for reducing oversight gaps and improving biosecurity resilience.

### 4.3 Cyberbiosecurity and data governance

As African institutions increasingly rely on external infrastructures for diagnostic support, surveillance and research, the risk for cyberbiosecurity incidents expands, ranging from unauthorized access to sensitive biological data to manipulation of digital design files or cyber-physical interference with automated laboratory systems (Frascarelli et al., 2023; Okon et al., 2025).

In many countries, data protection and cybersecurity regulations exist but are not tailored to local contexts. Fragmented or outdated ICT infrastructure, reliance on external cloud service providers further complicate the development of internally monitored systems. Without explicit requirements for secure data governance, the risk of data leakage, unauthorized synthesis, or compromise of critical laboratory functions increases (Kaur et al., 2023; Jørgensen and Ma, 2025). These challenges are further shaped by uneven and evolving AI governance frameworks. A recent analysis of AI regulation in healthcare across multiple African countries shows that governance is often fragmented, with AI oversight embedded within broader data protection, digital health, or consumer protection laws rather than addressed through dedicated local regulatory mechanisms (Hynek, 2026). This fragmented regulatory landscape may limit coordinated oversight of AI-enabled systems and complicate their integration into existing governance structures.

## 5 Governance priorities for managing SynBio-AI convergence in Africa

Through the analysis made on the different country strategies and continental biosafety and biosecurity bodies different recommendations were identified to address the governance gaps. It is worth noting that addressing these governance gaps identified does not require the creation of new regulatory bodies or resource-intensive institutions but an improvement on the already existing strategies. In many African countries, oversight systems are already established but operate within constrained budgets and limited technical capacity (Munga and Quansah, 2025). The priority, therefore, is aligning existing biosafety, research governance and science policy mechanisms with the realities of AI-enabled biological design (Wang et al., 2026). The following governance priorities are suggested to be operationally feasible and adaptable to diverse institutional environments.

### 5.1 Implementing proportionate and risk-based oversight

A first reform is to incorporate AI-use disclosure into established biosafety and ethics review processes. Institutional biosafety committees and research ethics boards can revise application templates to require investigators to indicate whether AI tools were used in experimental design, sequence optimisation, or predictive modelling (Samori et al., 2023). Similarly, national research funding agencies can introduce AI-bio risk considerations into grant evaluation criteria. Applicants proposing AI-assisted biological work should outline potential misuse risks and describe corresponding mitigation strategies. These measures require administrative modification and can be implemented within current institutional mandates. By increasing transparency at the proposal stage, oversight bodies gain visibility into digitally mediated workflows that may otherwise remain unexamined (De Haro, 2024). This recommendation can also be applicable in developing tiered oversight approaches. A tiered framework enables authorities to differentiate between low-risk research activities and higher-concern combinations of biological agents and advanced AI capabilities. Relevant criteria may include the risk classification of the organism involved, the degree of autonomy or generative capacity of the AI tool, whether DNA synthesis is internally managed or externally outsource and the containment level of the laboratory environment (Gillum and Moritz, 2025). Low-risk applications such as AI-supported analysis of non-pathogenic organisms should proceed with minimal additional administrative burden. Higher-risk scenarios, particularly those involving regulated pathogens and advanced design systems, would warrant enhanced review and documentation requirements.

### 5.2 Lack of cross disciplinary capacity

A recurring theme across national and regional assessments is the limited number of certified biosafety and biosecurity professionals in the region (Victor, 2025). While training programs and professional networks are expanding, many laboratories still rely on part-time or informally trained staff for biosafety and biosecurity functions and there are relatively few structured career pathways for biosafety or biosecurity officers. The emergence of SynBio-AI adds new and urgent competency requirements, including understanding of computational biology, AI-assisted sequence design and optimization tools, DNA synthesis screening principles, bioinformatics security and cyberbiosecurity practices. The need for transdisciplinary personnel that will be able to link SynBio, AI and biosafety has been emphasised as a need to overcome the technical challenge of the fragmented knowledge in these fields (Undheim, 2024). Current training curricula and professional development programs in Africa rarely cover these combined domains systematically, constraining institutions’ ability to recognize and assess risk patterns. Table 3 indicates priority skill areas that would be essential in governing the application of AI-Synbio synergy especially in the African region.

**TABLE 3:** Priority skills areas required for Syn-Bio and AI.

Skill area	Description	Potential target group	References
AI literacy in biological research	Foundational understanding of how AI tools are used in biological research, including their capabilities, limitations, and potential misuse pathways. Enables oversight bodies to evaluate AI-assisted research beyond traditional laboratory parameters	Biosafety committees, regulators, policymakers, ethics boards	Wheeler (2025), Kayaalp et al. (2025)
Computational biology	Practical ability to interpret sequence data, understand AI-assisted design outputs, and engage with biological databases and modelling tools	Researchers, biosafety officers, laboratory managers	Alley et al. (2019), Simon (2025)
Bio-digital risk assessment	Ability to identify and evaluate risks emerging from the intersection of AI and biological systems, including dual-use concerns, design-phase risks, and integration across digital and laboratory workflows	Biosafety reviewers, regulators, national focal points	Wheeler (2025), Whisnant and Dölken (2026), Kayaalp et al. (2025)

### 5.3 Establishing baseline expectations for DNA synthesis screening

In the last decade there has been a rapid move from wet lab to computational biology, making it more necessary to govern the systems involved (De Haro, 2024; Gillum and Moritz, 2025). DNA synthesis screening remains a critical preventive control in synthetic biology workflows. Although many commercial providers implement screening practices, standards may vary (Kane and Parker, 2024). At the regional level, scientific and regulatory bodies may develop harmonized minimum expectations regarding supplier screening practices. Establishing such baselines reduces reliance on heterogeneous external standards and reinforces preventive safeguards at a pivotal stage of the design–build process (Simon, 2025). Other preventive synthetic biology mechanisms have been proposed to avoid genetic transfers and safeguard biosafety and biosecurity such as genetic safeguards, DNA barcodes and genetic firewalls such as xenobiology (Wang and Zhang, 2019). Although these are forward looking measures that involve genetic engineering it is important to note that some African countries still have restrictions on genetic modifications of organisms which may hinder the progress of the afore mentioned precautionary measures. An important observation emerging from the governance review is the limited regulatory attention given to DNA synthesis screening. Although the reviewed governance intstruments (Table 1) have developed legislative and institutional regulations for governing biotechnology and genetically modified organisms, explicit requirements for screening synthetic DNA orders or establishing minimum screening standards for commercial providers were not identified. Consequently, existing oversight remains focused on downstream research activities, with comparatively little attention paid to upstream stages of the synthetic biology workflow.

On the other hand there has been developments on improving DNA synthesis screening processes and to try and make them region specific as much as possible. For example the International Biosecurity and Biosafety Initiative for Science (IBBIS) recently launched the DNA synthesis screening consortium (Wheeler et al., 2024). This initiative is aimed at peventing the fragmented landscape on DNA synthesis screening globally. This initiative also begun an African workstream consortium that will assist in strengthenin international coordination that will promote the screening standards. Through initiatives like this, varying DNA synthesis screening practices can be avoided.

### 5.4 Governance strategy for African nations

**FIGURE 1:**
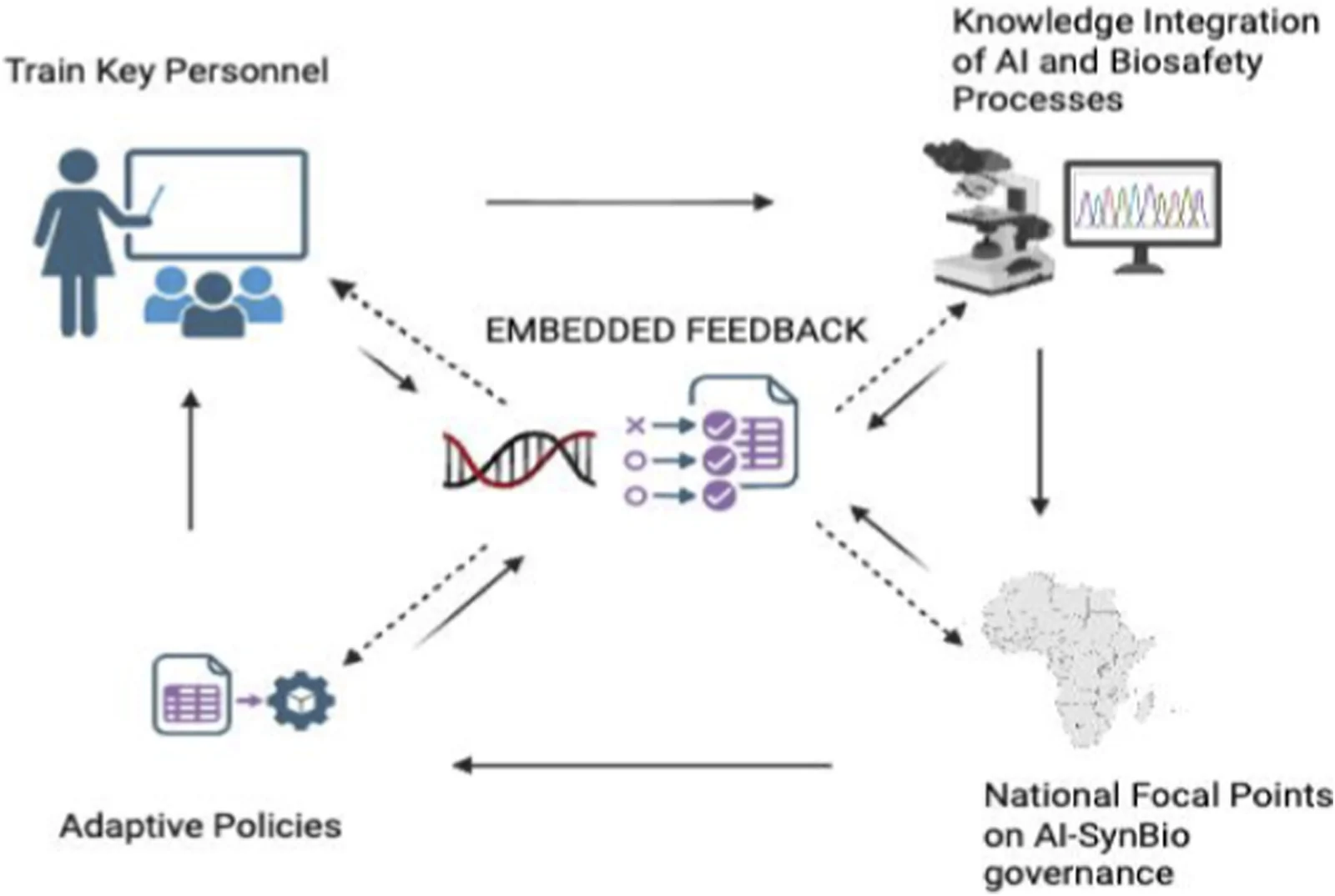
An adaptive governance approach for AI-Sybio convergence in Africa.

Based on the afrorementioned gaps and proposed solutions we suggest a governance model (Figure 1) that will involve improving on already existing workflows and introduce an embedded feedback loop for managing risks posed by AI and SynBio. Rather than relying on the expansion of regulatory institutions, the model strengthens governance by building capability, integrating oversight systems, assigning clear coordination roles and continuously adapting policy through feedback. The model operates through four interconnected functions: 1. Training key personnel across biosafety, digital governance and research systems; 2. Integrating AI considerations into existing review and funding mechanisms; 3. Establishing AI assited biology focal points to coordinate oversight across institutional boundaries; and 4. Adapting governance through a structured feedback intelligence layer that draws on biosafety reviews, procurement patterns and incident reporting.

## 6 Current standing and gaps of regional frameworks

Regional bodies such as the African Union and Africa CDC have begun to articulate comprehensive frameworks for health security, laboratory systems, and pandemic preparedness that serve as platforms for addressing emerging biorisk management challenges (Maruta et al., 2023). These frameworks emphasize integrated surveillance, standardized laboratory quality systems and accreditation, regional collaboration for outbreak detection and response, and capacity strengthening for health security. Variability in legal implementation, resource constraints, competing health and development priorities, and limited access to technical expertise mean that SynBio-AI risks are often not explicitly considered when translating global norms into national practice (Mutesi et al., 2025). Regional platforms convened by the AU and Africa CDC could therefore play a key convening and coordination role in translating global principles into practical tools, model policies and peer-learning mechanisms adapted to diverse African health and research systems. Policymakers and practitioners in the region are therefore at the forefront of curbing problems that may be caused by the AI in biological spaces and we recommend the following.

### 6.1 Integrate SynBio-AI into existing biosafety and biosecurity frameworks

Rather than creating entirely separate regulatory regimes for synthetic biology and AI, countries can incrementally incorporate SynBio-AI considerations into existing biosafety, biosecurity, and research governance instruments (Zeng et al., 2022). This approach will assist existing institutional capacity and authority while extending their scope. Specific actions include.

- Explicitly referencing synthetic biology, AI-enabled design tools and dual-use risks in revised national biosafety and biosecurity policies and strategic plans.
- Clarifying how dual-use risk assessment should be conducted for computational biology, AI-assisted design projects, and SynBio-AI research.
- Providing training and guidance to committee members and reviewers on recognizing and evaluating SynBio-AI dual-use concerns.
- Investing in cyber-biosecurity and DNA synthesis screening by implementing access logging and auditability for critical laboratory information systems and bioinformatics pipelines to detect unauthorized access or manipulation.
- Developing or adopting DNA synthesis screening practices, whether through national regulation, industry self-governance, or regional standards, to mitigate the risk of unauthorized synthesis of harmful sequences while preserving access for legitimate research, diagnostics, and innovation.

### 6.2 Professionalize and strengthen the SynBio-AI biosecurity workforce

Regional biosafety and biosecurity professional networks should co-develop competency frameworks and curricula for biosafety and biosecurity professionals that explicitly include SynBio-AI technologies, dual-use assessment, bioinformatics security, and cyberbiosecurity (Ayana et al., 2024). Strategic actions include.

- Establishing or updating professional competency frameworks for biosafety and biosecurity officers that reflect SynBio-AI skills and knowledge areas.
- Integrating SynBio, AI and dual-use governance into existing biosafety training programs, including those recognized by international or regional biosafety associations.
- Recognizing and supporting biosafety and biosecurity positions as core professional roles in laboratory, regulatory, and research organizations, with adequate compensation, career advancement, and resources.
- Creating a regional knowledge repository (online platform, policy library) that compiles country policies, guidelines, case studies, and lessons learned on SynBio-AI governance, accessible to African practitioners and policymakers (Gillum and Moritz, 2025; Owor et al., 2025).

## 7 Conclusion

The review undertaken in this study shows that many African countries already possess legislative and institutional mechanisms relevant to biotechnology, public health, biosafety, cybersecurity, and, increasingly, artificial intelligence. It also highlights that the growing use of synthetic biology and artificial intelligence presents a new set of governance questions that existing biosafety and biosecurity systems were not originally designed to address. Rather than revealing a complete absence of governance, the analysis points to recurring institutional conditions that are likely to influence how emerging approaches to AI-Synbio oversight can be implemented. Current international discussions increasingly focus on new governance approaches for AI-enabled biology, yet their implementation will inevitably depend on the institutional arrangements through which oversight is exercised. In the review, the principal challenge is seen to be enabling existing institutions to respond more coherently to technologies that increasingly cut across disciplinary and regulatory boundaries. The priorities identified in this paper build on that premise. Integrating AI considerations into established biosafety review processes, strengthening regulatory expertise at the interface of biology and digital technologies, improving coordination between institutions with complementary mandates and developing regional approaches to DNA synthesis screening represent practical steps that can be incorporated within existing governance arrangements. Such measures are likely to be more feasible and sustainable than establishing parallel regulatory structures for technologies that continue to evolve rapidly. As research with the use of biology and artificial intelligence will continue to expand, the effectiveness of governance will depend on the capacity of existing institutions to interpret, coordinate and apply them in practice. Understanding these institutional conditions is therefore essential if future governance efforts in the region are to keep pace with scientific advances while remaining responsive to national priorities and regional realities.
